# Periscope

**Published:** 1890-12

**Authors:** 


### PERISCOPE.

### MEDICINE.

**Notes on a Mode of Treatment by Vaccination of Experimental Tuberculosis** by M. Granchet and H. Martin.—In view of the publication of his experiments on inoculation for tuberculosis by Professor Koch, the authors think it advisable to publish their own experiments.

Rabbits were the animals chosen for the experiments, as in them artificial tuberculosis, with constant easily-recognised lesions, can be induced with certainty. Their plan was to inoculate rabbits with a given quantity of vaccine in the vein of the ear, taking some in which artificial tuberculosis had been induced, and others free from tuberculosis in order to control the results. Their researches extend over two years. They employed vaccines of varying strength, down to those whose virulence was *nil*, and so formed a rough scale of degrees of virulence, after the method of M. Pasteur. They called the most powerful vaccine, which kills a rabbit in from fifteen days to a month, No. 1; and the others, Nos. 2, 3, 4, 5, 6, 7, 8, 9, 10, according to the degree of virulence. Nos. 2 and 3 are always fatal, but after an interval of time varying according to the resistance offered by the animal. Five rabbits, on August 27th, were inoculated each with a half-syringe of strength No. 6; on 3rd September, and again on 12th with No. 6; then on 26th September with No. 2, and on October 15th with No. 1. Three rabbits, as a control experiment, were also on October 15th inoculated with No. 1, and died from 28th October to November 5th with classical lesions of tuberculosis. Of the vaccinated animals, three died in the same time as these last, and with the same lesions; but one survived to December 17th, and the other to January 7th, 1890, and only slight tuberculous lesions were found. The authors then made a new series of inoculations, with more frequent vaccinations with the weaker vaccines, stopping altogether with No. 2. They obtained results from this which they considered showed a more doubtful immunity from the disease, as the time for which healthy rabbits survive after inoculation with No. 2 varies much. In a last series, therefore, they vaccinated eleven rabbits with Nos. 6, 5, 4, 3, and 2, from January 30th to March 25th, 1890; and on April 10th, with No. 1: two healthy rabbits were also inoculated with No. 1 on this day, for control. The two latter died on May 3rd and 10th. Of the other eleven, two died on 16th and 26th June; two on 7th and 29th July; four on 4th, 7th, and 9th August; three are still alive, more than three months after the most virulent inoculation. They believe that their results show that by their vaccinations rabbits obtain a prolonged power of resistance against experimental tuberculosis, and an immunity from the effects of the disease, of which the duration remains still to be determined. The nature of the vaccine is not stated.—*Gazette des hôpitaux*, August 21st, 1890. J. M. C.

**Varices of the Œsophagus and Hæmatemesis in Chronic Alcoholism.**—At a meeting of the Sociétè Médicale des Hôpitaux, M. Letulle related the case of a man of

40 who suffered from hæmatemesis recurring about every two months, preceded by pains radiating from the stomach to the arms, and a general feeling of malaise. The blood gushed from the mouth and nose, was quite pure and unmixed with food. The spleen was large; the liver of normal size. The diagnosis of atrophic cirrhosis, with œsophageal varix, was made. After some months the patient had pleuritic effusion on the right side, and died of a very large hæmorrhage. The autopsy disclosed slight fatty degeneration in the liver; a very large "cirrhotic" spleen; some peritonitis; thrombosis of the superior and inferior mesenteric veins, and enormous varicose veins of cardiac end of œsophagus. In his remarks on the case, M. Letulle said that most instances of œsophageal varicose veins occurred in cases of chronic alcoholism; their mechanism, however, had not been thoroughly elucidated. Atrophy of the liver was not a constant lesion in these cases, and in many of them the state of the peritoneum and great intestinal veins was not noted. M. Debove said that it was well-known that in atrophic cirrhosis, hæmorrhage from the stomach or intestine was a frequent and formidable complication; moreover, it often occurred before the production of ascites, at a period of the disease when the diagnosis was difficult. He did not think that the obstacle to the circulation through the liver was the cause—at any rate, it was not the sole cause—of these hæmorrhages, for they did not occur after closure of the great venous trunks by a ligature or by a clot; and in the course of atrophic cirrhosis, they often occurred in the pre-ascitic period, when the hindrance to the portal circulation was not at its maximum. Can alcohol cause directly morbid changes in the gastric and intestinal capillaries? There is no certain evidence of this. In only three out of the very many cases of this class observed by M. Debove was there rupture of œsophageal varices; their lesion, then, is probably rare. He considered that the most probable cause of hæmorrhage in cases of cirrhosis was an intense congestion of the portal system. In the maintenance of the blood at an almost constant pressure in the arterial system, one of the most important means of counteracting the various influences which tend to cause it to vary lies in the regulating apparatus provided by certain parts of the venous system, which are capable of storing large quantities of fluid. The portal system of veins pre-eminently acts in this way, as the researches of Ludwig and Cyon have shown. Under vasomotor influences, a more intense congestion can be observed in the portal than in any other venous system. In a healthy man this congestion does not give rise to hæmorrhage, because the liver can hold a very large quantity of blood, and the passage of the blood into the vena cava is easy. This the liver can no longer accomplish in cirrhosis, and an intense congestion of the portal system will then be followed by hæmorrhage. These hæmorrhages occur less often at an advanced stage of cirrhosis, probably because ascitic fluid exerts a counter-pressure on the veins, and the patients are, moreover, very anæmic. The ruptures take place in the capillaries of the mucous membrane, and on account of their number and small size cannot be detected at the autopsy. Should there be a *locus minoris resistentiæ*, as in the case where œsophageal varices exist, rupture will occur there; under these circumstances, the trunk of the portal vein itself has been found ruptured in two or three cases, reported by Frerichs and by Wilks and Moxon.—*L'Union médicale*, October 23rd, 1890. J. M. C.

**Pneumonia after Injury.**—The following case, related by Minossi, illustrates how slight an injury may lead to pneumonia, as well as the readiness of the disease to recur. It is, moreover, remarkable for the very early recurrence, and shows that the opinion about the disease conferring a temporary immunity on the individual is not always warranted. The patient, a man of 54, was admitted into hospital on October 26th, 1888, suffering from a right pneumonia, which he "went through" well, and on the 19th November he was to have been discharged. On the morning of that day, while dressing, he was seized with giddiness and fell a short distance, striking the right side of his chest against the iron frame of the next bedstead. Some of his fellow-patients raised him and put him to bed, and during the remainder of the day he complained of nothing more than a slight pain in his right side. On the 20th the temperature rose, and the sputum became rusty. An accurate examination was made on the 23rd, when the expectoration was found viscid and rusty; temperature, 101·6°; pulse, 120 running; respirations, 36. Anteriorly, the note was tympanitic on percussion on the right side, with numerous crepitations and crepitant râles on auscultation. On the left, the breathing was harsh. Behind, on the right, respiration was imperceptible at the base, and the vocal fremitus weakened; above, it was increased, and rhonchi heard. On the 24th the general state was worse, and the following morning patient complained of pain in the left side, anteriorly. He died on the 26th. At the autopsy, no marks of contusion were found on the skin of the thorax. Effusion was found in both pleural cavities, and in the pericardium it was tinged with pus. The lower lobe of right lung was compressed somewhat by exudation, and adherent to chest wall. Just above this, corresponding to the point where the chest was struck, the parietal pleura was hyperæmic, with hæmorrhages. The whole upper and part of the lower lobe was in a state of croupous pneumonia. On the left, acute pleurisy, and lung œdema with recent pericarditis, were present.—*La Riforma Medica*, No. 148, 1890. D. R. P.

**Surgical Dressings.**—At the Tenth International Medical Congress, Professor Bergmann communicated to the members of the Surgical Section the following account of his ordinary method of dressings:—For two years Bergmann has exclusively used the aseptic in place of the earlier antiseptic methods in operations. This is based on the ascertained fact that the infection of wounds occurs through the air in very few cases only. The dust which falls from the air, and contains germs, only comes into contact with wounds for a very short time, and does, therefore, generally no harm. Besides, the surface of wounds can be protected from atmospheric dust by covering it with compresses as much as the operation permits. In order that the operation-room should contain as little dust as possible, the walls should be quite smooth and easily purified. This is not the case in the Berlin Clinic; for it was built at a period when the importance of atmospheric dust on the infection of wounds was not understood. The boards of the room are kept constantly damp, so that the particles falling from purulent wounds may not rise into the air, since it has been proved that from moist surfaces no microbes ascend.

Every care is taken that no infection of the wounds occurs by direct contact. To this end it is ordered that—

1. The skin of the patient be purified before the operation, both over the place where the wound will be made and for a considerable distance around it, in the following way:

The skin is first lathered and shaved. Next it is again thoroughly washed and lathered with sterilized water and glycerine-potash soap. It is then dried and thoroughly rubbed with a sterilized towel. The Professor insists on the importance of this rubbing being thoroughly and sufficiently carried out, because by it the upper layers of the epidermis are removed, on and in which lie the impurities which contain pathogenic microbes. Next the skin is washed with 80 per cent. alcohol, and, finally, with a 1 in 2,000 solution of sublimate.

2. The hands of the operator and assistants are purified and disinfected in exactly the same manner. Brushes which are kept in a 1 in 2,000 sublimate solution are used to purify the hands. Bacterial experiments show that brushes which lie exposed on the washstand contain great quantities of bacteria; therefore, they must be kept in solution.

3. Before each operation, the patient is laid on a dry cloth which has been sterilized, and is covered with a similar cloth, so that only the region which is to be operated on remains uncovered and accessible.

4. The instruments before use are boiled in a one-per-cent. solution of carbonate of soda for five minutes, in a vessel prepared for them, and which stands in the operation-room. In this they remain until they are used for the operation. If they become impure during the operation, it is sufficient to dip them for some seconds in the boiling soda-solution, which stands ready, in order to make them again pure and sterile.

In this way he succeeds in keeping them, not only perfectly sterile, but also sharp and free from rust. The visitors to the Clinic will have the opportunity to convince themselves of the advantages of this procedure.

5. During operation the bleeding is attended to with the greatest care. No sponges, but pads of previously sterilized wool are used for this. They are soaked in no antiseptic, but pressed dry on the wound and then thrown away. All vessels are secured with torsion forceps or Pean's clamps, and tied with catgut. The wound is not closed with stitches until perfectly dry and not a drop of blood is escaping. The gut threads are prepared from the catgut of the shops by being rolled in a single layer round thin plates of glass, and then laid in a five-per-cent. alcoholic solution of sublimate. The solution is at first renewed frequently, till it remains clear. The glass slips, with the threads, remain lying in it until shortly before use, when they are steeped in a simple one-per-cent. solution.

6. The silk employed for sutures is not sterilized in antiseptic solutions, but in steam.

7. The essential conditions for aseptic operations are the sterilization of all surroundings which come into contact with the operator, the assistants, and the patient. To this end, all towels in the operation-room, as well as the cloths upon which the patient lies and the linen overalls which the operators put on, are placed, shortly before the operation, in a disinfecting apparatus of Rietschel and Henneberg's make. In this sterilization is carried out by a jet of steam, after the principles developed by R. Koch. Such an apparatus is placed in the pavilion adjoining the operating-room, and its action will be explained to visitors. An electrical thermometer, placed in the middle of the linen in the apparatus, shows the moment when the steam, which rushes through it with great rapidity, reaches the temperature of 100° Cent. The articles remain half an hour from that moment in the chamber, when all organic particles and microbes in them are killed. The stuff is then perfectly sterile.

8. Exactly in the same way, all dressings are made germ-free in the same apparatus. The recent ones have been obtained from Böhm's factory (75 Oranienburger Strasse, Berlin). They are cut into pieces of the form and size for operations and dressings, and next placed in linen bags. The bags are tied up and put into the disinfecting apparatus. The wadding and roller-bandages are treated in the same way. All these stuffs remain in the bags, and the bags inside the disinfecting chamber until they are used. They are then taken out, laid on a sterilized towel, and covered with another on the operation-table. The attendants who take them from the bags have previously disinfected their hands in the previously described manner.

When purulent and tuberculous bones and joints are operated on the method employed in the clinic is, on the whole, the same. There is the same purification and disinfection of the region where the operation is performed; but the wounds are not closed immediately after the operation, but are filled with iodoform gauze, *i.e.* a soft dressing material into which iodoform powder has been rubbed. This remains in the wounds from one to two days; it is then removed, and the wounds, now first after forty-eight hours, are closed by sutures. These wounds heal by first intention without pus, though they are kept through two days plugged with iodoform gauze.—*Deutsche Medicinische Wochenschrift*, Aug. 7th, 1890.

G. P.

### THERAPEUTICS.

**Salol in Enteric Fever.**—M. Hirtz has employed salol to produce intestinal antisepsis. In enteric fever he gives a dose of ʒj. in the twenty-four hours, with salicylate of bismuth. He thinks that by this medication intestinal antisepsis is better attained than by naphthol, and it has the advantages over the latter that it is well borne by the patient and also disinfects the urinary tract. In the intestine salol is decomposed into phenic and salicylic acids, which are powerful antiseptics and antipyretics. He gave salol in daily dose of ʒjss. for three weeks to one patient, without causing any gastric disturbance. Given with bicarbonate of soda he has found salol useful in gastric ectasia.—*L'Union médicale*, Nov. 9th, 1890.

[I have found that naphthol or hydronaphthol is well borne by nearly all patients, and effectively produces intestinal antisepsis when administered according to the plan given in *The Practitioner*, July, 1890.] J. M. C.

**Strychnine in Cerebral Affections.**—From a series of pharmacological experiments, Dr. E. Biernacki has arrived at the following results:—1. Strychnine acts on the brain, producing diminution of cerebral excitability. 2. It is doubtful whether strychnine produces a direct influence upon the grey substance of the cortex; the decrease in excitability is probably called forth by the irritability of the cord, and perhaps, also, of other parts of the nervous system. 3. Favourable results of strychnine in dipsomania and sleeplessness may be explained by this action. The alkaloid is, as already known, of great service in dipsomania and as a palliative in epilepsy of the cortical form. In both cases it had been used empirically, and no explanation given. More recently Lauder Brunton has advocated strychnine as a hypnotic in sleeplessness caused by strain of the nervous system, where opium and other narcotics gave no good results; bromide of potassium was without effect; and in one instance, after chloral, he observed a maniacal condition. Strychnine acts best in that state in small doses, $\frac{1}{100}$ gr.; or in form of the tincture of nux vomica, 5—10 drops. It was useful, likewise, in brain anæmia. Biernacki thinks it desirable that it should be tried therapeutically in different states of irritability of the cerebral cortex.—*Therapeutische Monatshefte*, No. 8, 1890.
D. R. P.

**Bromoform and Phenacetin in Whooping Cough.**—Bromoform was first used in whooping cough by Stepp, who claimed to have seen, in one hundred instances, recovery within two to four weeks. Dr. H. Neumann has recently tested the substance, having treated twenty-five out of sixty-one cases with it. The dose was a few drops (2—5) suspended in syrup, and no ill effects were seen. Where the drug was useful, this showed itself in a few days; the number of attacks being lessened in some cases, and in others the intensity of seizures diminishing—the latter effect being more frequently observed. It is not at all certain that bromoform can shorten the duration of the attack; for even with continuous and energetic administration, the convulsive stage lasted its usual time. Neumann's opinion of this treatment is, that it had mostly a good effect upon the seizures, and it might be recommended on account of its freedom from evil consequences and not unpleasant taste; but it is not more a specific for pertussis than quinine, antipyrin, and other substances. With these and other remedies, varying results are obtained in the same epidemic—some shorten attacks, others have no result.

Writing of Phenacetin, which has likewise been advocated, Neumann says that he gave it in nine cases, the quantities ranging from $\frac{1}{5}$ gr. to 3 grs. several times daily. In a girl seven years old, suffering for three weeks from whooping cough, the attacks became at once lighter, shorter, and fewer in number, and got worse on intermitting the phenacetin; but in the other instances no appreciable advantage was observed.—*Therapeutische Monatshefte*, No. 7, 1890.

D. R. P.

### OBSTETRICS.

**The Binder.**—To bind or not to bind the parturient female is a question with which the obstetrician still allows his mind to wrestle. At a recent meeting of the Obstetrical Society of London it was concluded in effect that the doctor had better do as the patient felt inclined. The matter ought not to be left in such a doubtful state. Kingdoms rise and fall, great nations disappear, cyclonic storms and volcanic fires change the face of nature, but babies continue to be born and the mother insists on having a binder, or knowing the reason why.

The Obstetrical Society of London should not trifle with topics of such eternal moment. For our part, we say do not bind. The alleged comfort secured is imaginary, the idea that the binder can restore the figure is unscientific and unphysiological, its supposed power in helping involution is purely hypothetical, and the whole conception of a binder is unnatural and abhorrent to common-sense.

The binder is a relic, not of barbarism, for barbarians are too sensible to bind, but of a half-enlightenment that is worse than ignorance. The physician who binds nowadays does it out of a weak complaisance to an imperious mother-in-law or officious nurse. He should, out of respect to his art, rise above such trammels to his medical skill and his intellectual independence.—*Medical Record*, May 31st, 1890.

